# The Impact of COVID-19 Pandemic on Psychiatric Emergencies in Two Different Settings: Emergency Room and Community Mental Health Service

**DOI:** 10.3389/fpsyt.2022.894939

**Published:** 2022-05-26

**Authors:** Rosaria Di Lorenzo, Margherita Pinelli, Davide Bertani, Anna Cutino, Diego Dragone, Claudia Elia, Riccardo Farina, Gianluca Fiore, Filippa Luisi, Sofia Panico, Laura Valeo, Sergio Rovesti, Paola Ferri

**Affiliations:** ^1^Service of Psychiatric Diagnosis and Care, Department of Mental Health and Pathological Addictions, AUSL Modena, Modena, Italy; ^2^Community Mental Health Service, Department of Mental Health and Pathological Addictions, AUSL Reggio Emilia, Reggio Emilia, Italy; ^3^School of Psychiatry, University of Modena and Reggio Emilia, Modena, Italy; ^4^School of Nursing, University of Modena and Reggio Emilia, Modena, Italy; ^5^Department of Biomedical, Metabolic and Neural Sciences, University of Modena and Reggio Emilia, Modena, Italy

**Keywords:** psychiatric emergencies, COVID-19 pandemic, Emergency Room, Community Mental Health Service, vulnerable people

## Abstract

**Background:**

The SARS-CoV-2 pandemic caused a public health emergency with profound consequences on physical and mental health of individuals. Emergency Rooms (ER) and Community Mental Health Services (CMHS) played a key role in the management of psychiatric emergencies during the pandemic. The purpose of the study was to evaluate urgent psychiatric consultations (UPCs) in the ERs of the General Hospitals and in the CMHS of a Northern Italian town during the pandemic period.

**Methods:**

This monocentric observational study collected UPCs carried out in ER from 01/03/2020 to 28/02/2021 (the so called “COVID-19 period”) and the demographic and clinical characteristics of patients who required UPCs in the 12-months period, comparing these data with those collected from 01/03/2019 to 29/02/2020 (the so called “pre-COVID-19 period”). The same variables were collected for UPCs carried out in CMHS from 01/03/2020 to 31/01/2021 and compared with those collected from 01/03/2019 to 31/01/2020. The data, were statistically analyzed through STATA 12-2011.

**Results:**

In ER, we reported a 24% reduction in UPCs during the COVID-19 period (*n* = 909) in comparison with the pre-COVID-19 period (*n* = 1,194). Differently, we observed an increase of 4% in UPCs carried out in CMHS during the COVID-19 period (*n* = 1,214) in comparison with the previous period (*n* = 1,162). We observed an increase of UPCs in ER required by people who lived in psychiatric facilities or with disability pension whereas more UPCs in CMHS were required by older people or those living in other institutions compared to the previous period. In the COVID-19 period, the most frequent reasons for UPCs in ER were aggressiveness, socio-environmental maladjustment and psychiatric symptoms in organic disorders whereas in CMHS we reported an increase of UPCs for control of psychopharmacology therapy and mixed state/mania.

**Conclusion:**

In light of our findings, we conclude that the most vulnerable people required more frequent attention and care in both ER and CMHS during pandemic, which disrupted individuals’ ability to adapt and induced many stressful reactive symptoms. In order to reduce the impact of the COVID-19 pandemic on mental health, psychological support interventions for the general population should be implemented, having particular regard for more psychologically fragile people.

## Introduction

Since ancient times, humankind has faced pandemic infections which decimated entire populations, changing the course of history. The COVID-19 pandemic rapidly spread during late 2019 and early 2020; the pathogenic agent is a virus called SARS-CoV-2, which belongs to the coronavirus family and is responsible for a potentially very severe acute respiratory syndrome (1). Starting from China, cases of the disease then quickly spread to other continents. On January 30, 2020, the World Health Organization (WHO) declared the sickness caused by the new coronavirus to be a public health emergency of international concern (2). On March 11, 2020, COVID-19 disease was officially declared a pandemic (3, 4), the meaning of which refers to the spreading of a disease over large regions of the world or the entire planet Psychiatry of catastrophes. Exposure to disasters is a major risk factor for the development of mental illness with a frequency, which can range from 8.6 to 57.3% (5). Some clinical manifestations may fade over time, while others may require early, targeted intervention (6). WHO stated that the COVID-19 pandemic is not only a potential global epidemic risk, but also a danger for exacerbation, development and relapse of many psychiatric and substance abuse disorders (7).

In the immediate aftermath of the COVID-19 pandemic onset, most of the general population reported negative psychological symptoms such as anxiety, depression, stress, insomnia, phobias, and compulsive behavior; the most negative impact was found in the female gender and young adults (8). The first large studies carried out on the Chinese population at the beginning of the pandemic showed that substantial portions of the population (35–54%) presented psychological distress quantified by themselves from moderate to severe; the symptoms most represented were those of the anxiety spectrum, followed by depression, phobias, compulsive and avoidant behaviors, impaired social functioning (9, 10) and Post-Traumatic Stress Disorder (PTSD) with a prevalence of 18.4%, especially in people who were most exposed to the infection (11). Quarantine and lockdown generated feelings of boredom, anger, uncertainty, frustration, irritability, increased suicidal thoughts, aggressive behavior and sleep disturbances (12–14). During these phases of the pandemic, actual hoarding of basic necessities and health care products occurred in the general population, enhanced by panic and unwarranted fear about resource scarcity (15). A secondary effect of the prolonged lockdown was the collapse of many public services, as well as the shutdown of businesses and industries: many people lost their jobs and faced severe financial crises, which further contributed to intensifying the negative emotions that arose with the onset of the pandemic (16). In some cases, house isolation was related to increased addictive behavior, including substance use (17), and in others, it reportedly contributed to escalating family tensions and violence within the household, especially in contexts already marked by domestic abuses (18).

The main psychological consequences brought about by the COVID-19 pandemic were the increase of depression, anxiety and acute stress disorders, as suggested by most authors (19–22). Notably, these disorders primarily occurred in people who had already been diagnosed with a psychiatric disorder prior to the pandemic, thus being, overall, more vulnerable. One study found that at least 50% of psychiatric patients reported a worsening of their psychopathological conditions (23). Patients with chronic psychiatric conditions who lived in residential facilities found themselves confined to these environments, with no opportunity for movement or social interactions (24).

The COVID-19 pandemic significantly affected how mental health care is provided and delivered, impacting access to care at first and, consequently, the quality of services provided (25–27). During the COVID-19 pandemic, one of the first goals of the healthcare organization was to ensure people’s safety, especially through social distancing; telemedicine was able to safely connect physicians and patients, granting access and continuity of care (28, 29).

Despite the negative impact of the pandemic on mental health, an overall decrease in the total urgent territorial psychiatric consultations and hospitalizations were observed in the first months of 2020 compared to the same period in 2019 (30–35). In particular, a French study reported an approximately 50% decrease in consultations performed for psychiatric emergencies in three different psychiatric centers in Paris, with an increase of consultations for only psychotic disorders in the pandemic period compared to 2019 (36). The reduction in the number of psychiatric consultations in outpatient services during the first period of 2020 compared with the same period in 2019 was also confirmed by Italian studies (37, 38). A German study reports the reduction of psychiatric emergency consultations during March 2020 compared with March 2019, particularly for chronic affective psychiatric disorders (32). Overlapping results are also reported in other studies (27, 35). In any case, the initial decline in urgent psychiatric consultations has since been offset by a substantial increase beginning in May 2020, according to some studies (34, 39). This observation could be explained by reasons such as the fear of potential SARS-CoV-2 infection and the underestimation of psychological needs, due to the health priority given to COVID-19 disorders. Emergency psychiatric consultations have been readily replaced by telemedicine when possible in order to provide help to people in a safe mode (25, 34).

Many observations reported a general increase in depressive disorders during the pandemic (19–22), whereas other authors noticed an opposite trend (35, 40). This conflicting data can be explained by different reactions to the pandemic: some individuals, when faced with pandemic-related stress, presented a depressive reaction, while others may have experienced a clinical improvement, thanks to the reduction in external demands, resulting in a greater sense of stability and balance (35, 40). During the pandemic, many requests for psychiatric emergency care and assessment were due to symptoms related to anxiety disorders (41), which represented the major part of every consultation request (27). Notably, one study shows that, compared with 2019, emergency accesses of people with neurotic, stress-related, and somatoform symptoms increased in 2020, especially in males compared to females (35). A Spanish research group reported during the COVID-19 pandemic an increase of brief psychotic disorders reactive to the stressful scenario caused by the pandemic; this kind of disorder appears to be related to an increase in suicidal behavior and low diagnostic stability over time (42). An increase in admissions for acute psychosis was also reported by other studies (27, 43). An increase of psychiatric admissions was also found in patients diagnosed with personality disorder and many requests for urgent consultations were made mainly for behavioral alterations based on an emotional instability and extreme reactivity to the pandemic stress (27). A Spanish study monitored emergency department admissions for psychiatric emergencies-urgencies during the period from November 2018 to April 2020 (44). Focusing on the issue of emergency admissions for suicidal ideation or attempted suicide, the study shows a decrease of this condition during the first phase of the pandemic, probably due to a general reduction in admissions for psychiatric emergencies and not a real decrease in suicidal intent (44). In contrast, other recent studies have reported that during the first phase of lockdown the accesses for self-injurious ideation increased, even those with concrete intent and suicide planning (33, 39). An increase in consultations in the hospital setting for patients with autism spectrum disorder, obsessive-compulsive behaviors, and substance abuse (31) was observed by other researchers (37). An American study found that females were more likely to make an emergency visit for co-occurring anxiety symptoms and substance abuse, whereas males for isolated substance abuse (39). Individuals who required psychiatric urgent consultations during the lockdown had a more severe psychopathological clinical condition than those seen in the period immediately before (33). One study found that many accesses to the Emergency Department were made by the elderly, more vulnerable to isolation conditions than other populations (27). The forced confinement that followed the restrictive measures also led to the development in the elderly of a sense of estrangement responsible for the increase of anxiety symptoms (45). After an initial reduction of the total number of emergency consultations over all, one study pointed out cases of increased multiple visits for the same patient: this phenomenon could be due to the reduction in the number of hospitalizations or, in any case, the reduced use of hospitalization in cases of acute worsening of psychiatric conditions during the pandemic (35).

### Primary Objectives

To detect any change in requests for urgent psychiatric consultations carried out in the Emergency Room (ER) and Community Mental Health Service (CMHS) in a Northern Italian town during the period of COVID-19 pandemic and related measures of social distancing in Italy, with particular attention to the variation in terms of frequency and type of requests.

### Secondary Objectives

• To analyze the correlation between demographic, clinical, social and environmental risk factors and urgent psychiatric consultations in ER and CMHS, both in the period of social confinement and in the following months, and compare them with those collected in the corresponding months of the previous year.
• To analyze the interventions carried out in psychiatric emergency accesses in ER and CMHS, both in the period of social confinement and in the following months, comparing them with those carried out in the previous year to detect any differences.

## Materials and Methods

### Study Design, Setting and Period

The design of this observational study is monocentric, retrospective and comparative. It represents the continuation of two other studies implemented in the same settings which analyzed with the same design the psychiatric emergencies in a period of 6 months, from 1-3-2020 to 31-8-2020, compared with the same months of the previous year (38, 46). The settings are the Emergency Room (ER) of the General Hospitals and the Community Mental Health Service (CMHS) of a Northern Italian town, where urgent psychiatric consultations (UPCs) are carried out. The local Mental Health Department is organized into four sectors: Adult, Child and Adolescent, Pathological Addictions, Clinical Psychology. Community Mental Health Service (CMHS) provides diagnostics, therapy and rehabilitation treatments for adults in outpatient and inpatient services for a population of 703,203. It manages voluntary and compulsory hospitalizations to the public acute psychiatric ward (Service of Psychiatric Diagnosis and Care) and urgent psychiatric consultations at Emergency Room (ER) of General Hospitals.

A comparison was made between the UPCs carried out in ER during a period of 1 year, from 1 March 2020, the beginning of Italian lockdown, to 28 February 2021 and the consultations carried out in the same period of the previous year, from 1 March 2019 to 29 February 2020. A similar comparison was performed for the UPCs carried out in CMHS, with a slight period difference due to logistic difficulties in collecting data (the computer application was changed in February 2021, not allowing us to extract this information): a comparison was made between UPCs carried out in the period ranged from 1 March 2020 to 31 January 2021 and the consultations carried out in same period of the previous year, from 1 March 2019 to 31 January 2020. The time periods prior to March 1, 2020 are identified by the term “pre-COVID-19 period” and subsequent ones with the term “COVID-19 period.”

### The Samples Analyzed

The following variables were collected for the sample of UPCs carried out both in ER and CMHS:

• referral to UPC;
• clinical reasons for UPCs;
• clinical activities performed: therapeutic prescriptions and/or drug administration in UPC, supplementary diagnostic test and/or additional medical consultations, Short-Stay observation in ER (an access modality which permits patients to remain in ER for 24 h in order to undergo further medical examinations or to wait for the availability of a bed in a psychiatric unit);
• UPC outcomes;
• number of UPCs per month;
• number of UPCs per patient during the observation periods.

Only for the sample of urgent consultations carried out in CMHS, the variable “Setting” (CMHS, home visit, telephone contact, other as video-call), was collected.

The following variables were collected for the sample of subjects who required UPCs during the observation period:

• age;
• gender;
• nationality (Italian, European, Extra-European);
• housing (family of origin, acquired family, homeless, protected facility, community, alone, others);
• employment status (employed, unemployed, student, retired, disability pension);
• psychiatric diagnoses according to the classification system (ICD-9-CM) indicated by local guidelines (47);
• double diagnosis of substance/alcohol use;
• medical comorbidity;
• previous treatment and care in CMHS, Pathological Addiction Service (PAS) and other community outpatient services.

After having collected data, we analyzed the qualitative and quantitative differences between the pre-COVID-19 period and COVID-19 period. The analysis was conducted separately for the samples of UPCs carried out in ER and in CMHS. The information relating to the UPCs carried out at ER were extrapolated from the patient information system used at the General Hospitals and the information relating to the UPCs carried out at the CMHS were extrapolated from the InfoClin Web computer system, an application in use since 2000 for the collection of socio-demographic, clinical and pharmacological data of users in treatment at CMHS. This application has recently been changed. Therefore, data integration was only possible for subjects already in treatment at the CMHS before the outbreak of the COVID-19 pandemic.

### Sample Eligibility Criteria

The sample is represented by all subjects aged 18 or over who received UPC in ER of the two General Hospitals or in CMHS of a Northern Italian town during the observation periods. Incomplete consultations were excluded from the samples.

### Statistical Analysis

We used descriptive statistical analysis: mean and standard deviation, *t*-test for analysis of continuous variables; percentages and χ^2^ tests or Fisher’s exact test for categorical variables and subsequent evaluation of the Standardized Residues (SR) with > or < 2 values and statistical significance of *p* < 0.05. We performed a multiple logistic regression, stepwise backward and forward model, between the dependent variable “UPCs” (“UPCs in pre-COVID-19” = 0, “UPCs in COVID-19 period” = 1) and the other selected variables as independent ones. The level of statistical significance was set at *p* < 0.05. The data was analyzed through STATA-12-2011.

## Results

### Urgent Psychiatric Consultations During the Two Observation Periods

**FIGURE 1:**
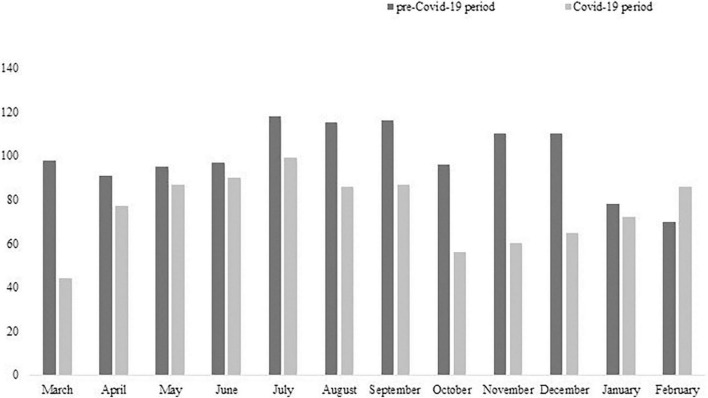
Number of UPCs per month in ER during the two observation periods.

**FIGURE 2:**
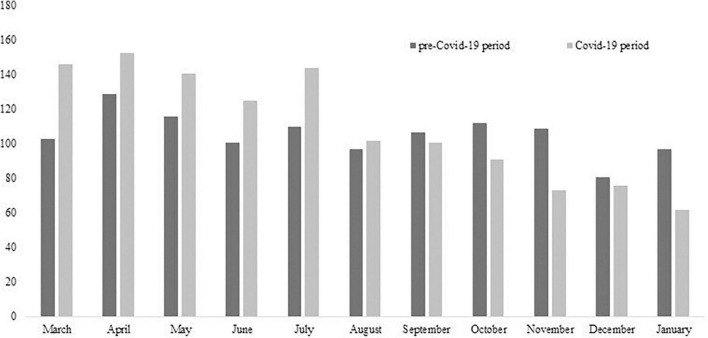
Number of UPCs per month in CMHS during the two observation periods.

In ER, we reported a 24% reduction in the number of UPCs during the COVID-19 period (*n* = 909) in comparison with the pre-COVID-19 period (*n* = 1,194). Differently, we observed an increase of 4% in UPCs carried out in CMHS during the COVID-19 period (*n* = 1,214) in comparison with the previous period (*n* = 1,162). The number of UPCs per month statistically significantly differed between the pre- and COVID-19 period both in ER (χ^2^ = 32.97, *p* = 0.001; Figure 1) and CMHS (χ^2^ = 35.33, *p* = 0.001; Figure 2) but in opposite ways. In particular, we observed a reduction of UPCs (*n* = 44) at ER in the month of March 2020 (the start of Italian lockdown due to the outbreak peak) in comparison with March 2019 (*n* = 98), whereas in the same month of 2020 the UPCs increased in CMHS (*n* = 146) in comparison with March of the previous year (*n* = 103). The total number of consultations carried out per patient in CMHS was higher in the COVID-19 period (*m* = 2.63 ± 2.66 SD) compared to the pre-COVID-19 period (*m* = 2.15 ± 2.22 SD) with a statistically significant difference (*t* = −4.77, *p* = 0.001, *t*-test). We did not report any significant difference in the number of UPCs per patient between the COVID-19 period (*m* = 1.31 ± 0.98 SD) and the pre-COVID-19 period (*m* = 1.33 ± 1.13 SD) in ER (Table 1). Among the subjects who required UPCs in ER, we observed that most of them came from home both in pre- (92.1%) and COVID-19 period (87.8%), with a significant increase of patients who lived in a psychiatric facility during the pandemic period (SR = 4.84, *p* < 0.05; Fisher’s exact = 0.000), as shown in Table 1. Similarly, in both the pre- and COVID-19 period, most of the subjects requiring UPCs at CMHS came from home (pre-COVID-19 period: 87%; post-COVID-19 period: 89%). However, we reported a statistically significant increase during pandemic period of patients who lived in community or protected facilities (SR = 4.31, *p* < 0.05; χ^2^ = 29.9; *p* = 0.000; Table 1). The settings of UPCs in CMHS significantly differed between the two periods (χ^2^ = 83.95; *p* = 0.000) due to the more prevalent telephone or video-call in the pandemic period compared to the previous period, as shown in Table 2. During the COVID-19 period at the CMHS, we highlighted a significant reduction in therapy administration during consultations compared to pre-COVID-19 period (χ^2^ = 52.38; *p* = 0.001), whereas we did not report any significant difference in clinical activities performed during UPCs in ER between the two observation periods (Table 2).

**TABLE 1:** UPCs in ER and CMHS during the two observation periods: number per patient, referral to and outcome.

Variables			UPCs in ER					UPCs in CMHS		
			Pre-COVID-19 period (*n* = 1,194)			COVID-19 period (*n* = 909)	Probability statistical test	Pre-COVID-19 period (*n* = 1,162)	COVID-19 period (*n* = 1,214)	Probability statistical test
**UPCs per patient, m ± DS**										
Number				1.33 ± 1.13		1.31 ± 0.98	NS	2.15 ± 2.22	2.63 ± 2.66	*t* = −4.77 *p* = 0.000
**Referral to UPCs, n (%)**										
Spontaneous/General practitioner				1100 (92.1%)		798 (87.8%)	Fisher’s exact = 0.000 ^§^ SR = 4.84	1008 (86.75%)	1076 (88.63%)	χ^2^ = 29.9, *p* = 0.000 *SR = 4.31
CMHS				4 (0.3%)		8 (0.9%)		4 (0.34%)	4 (0.33%)	
Other medical specialists				2 (0.2%)		1 (0.1%)		14 (1.2%)	21 (1.73%)	
Psychiatric facilities				32 (2.7%)		65 (7.2%)^§^		101 (8.69%)	68 (5.6%)	
Other institutions				46 (3.9%)		24 (2.6%)		5 (0.43%)	32 (2.64%)*	
Unknown				10 (0.8%)		13 (1.4%)		30 (2.58%)	13 (1.07%)	
**UPC Outcome, n (%)**										
Voluntary psychiatric hospitalization					313 (26.2%)	211 (23.2%)	χ^2^ = 53.99 *p* = 0.000 **SR = 3.99 ^§§^ SR = −2.17	109 (9.4%)	105 (8.6%)	χ^2^ = 19.84 *p* = 0.047 ***SR = 2.9
Involuntary psychiatric hospitalization					22 (1.8%)	12 (1.3%)		30 (2.6%)	30 (2.5%)	
Discharge at home/General practitioner					134 (11.2%)	76 (8.4%)^§§^		687 (59.1%)	758 (62.4%)***	
Referral to CMHS					493 (41.3%)	427 (47.0%)		225 (19.4%)	198 (16.3%)	
	PAS				63 (5.3%)	40 (4.4%)		16 (1.4%)	23 (1.9%)	
	Other Specialists				60 (5.0%)	62 (6.8%)		29 (2.5%)	20 (1.6%)	
	More than one outpatient service				37 (3.1%)	62 (6.8%)**		2 (0)	7 (1%)	
Others					63 (5.3%)	11 (1.2%)		31 (3%)	40 (3%)	
Unknown					9 (0.8%)	8 (0.9%)		19 (1.6%)	7 (0.6%)	
Referral to Home care					–	–		3 (0.3%)	16 (1.3%)	
		ER			–	–		5 (0.4%)	9 (0.7%)	
		Private specialist						11 (0.9%)	10 (0.8%)	
					–	–				
Self-discharge					–	–		26 (2.2%)	31 (2.6%)	

^§^, ^§§^, *, **, *** Standardized Residues (SR) with > or < 2 values and p < 0.05.

**TABLE 2:** UPCs in CMHS and ER during the two observation periods: setting and clinical activities.

Variables	Pre-COVID-19 period: UPCs in CMHS (*n* = 1,162) ER (*n* = 1,194)	COVID-19 period: UPCs in CMHS (*n* = 1,214) ER (*n* = 909)	Statistical test probability
**UPCs in CMHS: Setting, n (%)**			
CMHS	1006 (86.6%)	900 (74.1%)*	χ^2^ = 83.95 *p* = 0.000 *SR = −7.57 **SR = 3.03 ***SR = 8.23
Home patient	67 (5.8%)	81 (6.7%)	
Video call	62 (5.3%)	103 (8.5)**	
Telephone call	25 (2.2%)	126 (10.4%)***	
**UPCs in CMHS: Therapy administration, n (%)**			
Drugs administered	832 (72%)	708 (58%)	χ^2^ = 52.38 *p* = 0.001
No drugs administered	330 (28%)	506 (42%)	
**UPCs in ER: Supplementary diagnostic test and/or additional medical consultations, n (%)**			
Not required	1057 (87.9%)	791 (87%)	Not statistically significant
Required	137 (12.1%)	118 (13%)	
**UPCs in ER: Short –Stay observation**			
Not applied	1050 (87.9%)	806 (89.4%)	Not statistically significant
Applied	144 (12.1%)	96 (10.6%)	
**UPCs in ER: Therapy prescription and/or administration**			
No therapy prescription and/or administration	584 (49.9%)	411 (45.2%)	Not statistically significant
Therapy prescription	234 (19.6%)	222 (24.4%)	
Therapy administration	376 (31.5%)	276 (30.4%)	

^§^, ^§§^, *, **, *** Standardized Residues (SR) with > or < 2 values and p < 0.05.

### Clinical Reasons for Urgent Psychiatric Consultations

**FIGURE 3:**
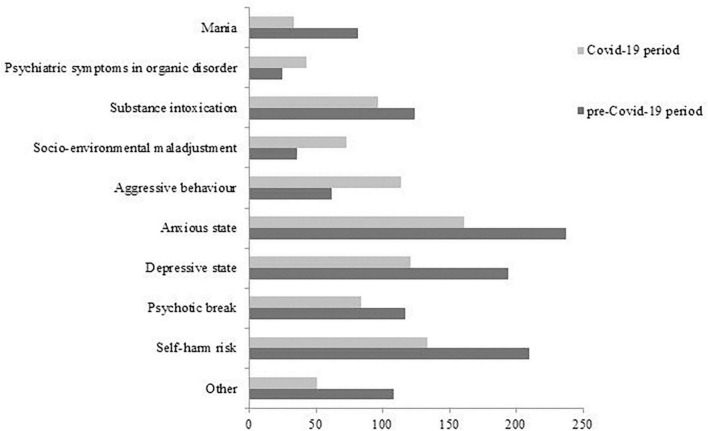
Clinical reasons for UPCs in ER during the two observation periods.

The clinical reasons for UPCs in ER, which we grouped into 10 main categories in accordance with the prevalence observed, statistically significantly differed between the two periods, as shown in Figure 3 (χ^2^ = 94.13; *p* = 0.001). In particular, three clinical categories were significantly prevalent during the COVID-19 period compared to the previous period:

1. aggressive behavior (SR = 6.03, *p* < 0.05) increased in May (SR = 2.09, *p* < 0.05), July (SR = 2.56, *p* < 0.05), November (SR = 3.61, *p* < 0.05) and December (SR = 2.74, *p* < 0.05),
2. socio-environmental maladjustment (SR = 5.14, *p* < 0.05) increased in April (SR = 3.15, *p* < 0.05), July (SR = 2.66, *p* < 0.05), September (SR = 2.33, *p* < 0.05) and December (SR = 4.37, *p* < 0.05).
3. psychiatric symptoms in organic disorders (SR = 3.39, *p* < 0.05) increased in June (SR = 2.03, *p* < 0.05) and November (SR = 2.12, *p* < 0.05).

**FIGURE 4:**
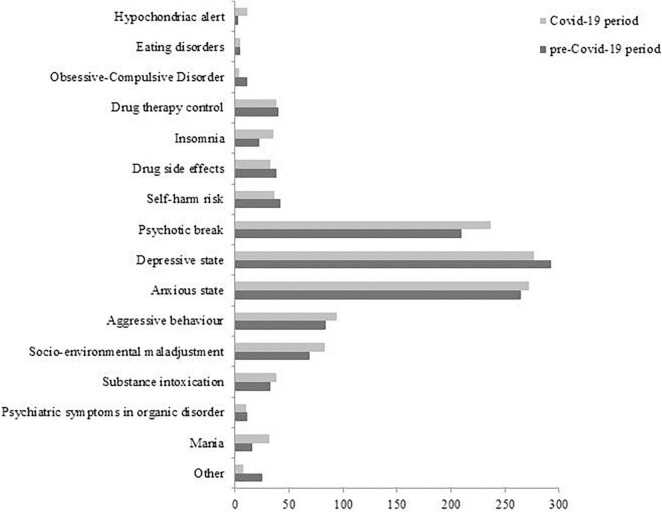
Clinical reasons for UPCs in CMHS in the two observation periods.

Regarding the CMHS, we grouped the most frequent clinical reasons for UPCs into 20 categories, whose frequency statistically significantly differed between the two periods of observation, as seen in Figure 4 (χ^2^ = 69.76; *p* = 0.001). In particular, the frequency of the following three clinical reasons for UPCs significantly differed in the two periods of observation:

1. drug therapy control (SR = 4.65, *p* < 0.05);
2. mania (SR = 2.12, *p* < 0.05);
3. anxious-depressive state (SR = − 4.15, *p* < 0.05).

In particular, during the COVID-19 period, in March we detected an increased UPC demand for insomnia (SR = 2.25, *p* < 0.05) and control of drug therapy (SR = 2.85, *p* < 0.05), while in November we reported an increased request for socio-environmental maladjustment (SR = 3.76, *p* < 0.05) and, at the same time, a reduction for anxious-depressive symptoms (SR = −2.50, *p* < 0.05).

### Outcome of Urgent Psychiatric Consultations

The outcomes of UPCs were significantly different between the pre- and COVID-19 period both in ER (χ^2^ = 53.99; *p* = 0.000) and CMHS (χ^2^ = 19.84; *p* = 0.047), as shown in Table 1. In particular, as regards the outcome of the UPCs carried out in ER, we observed a reduction of voluntary admissions as well as a decrease of the referral to General Practitioner (SR = −2.17, *p* < 0.05) whereas the compulsory admissions were not reduced and the referral to more than one service was increased (SR = 3.99, *p* < 0.05). The outcome of UPCs in CMHS which significantly increased during COVID-19 period was the referral to home and General Practitioner (SR = 2.9, *p* < 0.05). In the COVID-19 period, the outcome of UPCs in ER statistically significantly differed in the various months: in February, the prevalent outcome was the referral to CMHS (SR = 2.04, *p* < 0.05) and in September the referral to CMHS and other outpatient services (SR = 3.09, *p* < 0.05), while in December the sending home (SR = 2.25, *p* < 0.05) and to CMHS and other outpatient services (SR = 2.27, *p* < 0.05) were prevalent. Similarly, in the COVID-19 period, the prevalent outcome of UPCs in CMHS was the sending home (SR = 2.9, *p* < 0.05), especially increased in the month of June (SR = 3.88, *p* < 0.05).

### Subjects Who Required Urgent Psychiatric Consultation

The sample of subjects who required an UPC at ER consists of 1,598 subjects in the two observation periods, 896 in the pre-COVID-19 period and 702 in the COVID-19 period, whereas the subjects who required an UPC at CMHS were 1,265 in the two observation periods, 666 in the pre-COVID-19 period and 599 in the COVID-19 period. Regarding gender and nationality of the subjects visited in urgent consultation at ER and CMHS, any statistically significant difference between the two observation periods was highlighted (Table 3). We observed a significant difference between the two periods regarding the age of people who required an UPC only at CMHS: in the COVID-19 period they were older compared to those who needed UPC in the previous period (*t* = −2.42, *p* = 0.015, *t*-test) (Table 3). Regarding housing context and working activity of the subjects in our two samples, we reported significant differences between the two period only among people who required an UPC at ER, as reported in Table 3: patients who lived in a psychiatric facility (χ^2^ = 15.35, *p* = 0.018; SR = 2.41, *p* < 0.05) as well as subjects who were pensioners due to disability (χ^2^ = 29.27; *p* = 0.000; SR = 4.24, *p* < 0.05) required an UPC more frequently during the COVID-19 period. Among the UPCs carried out at CMHS, we did not highlight any statistically significant difference between the two periods regarding the housing and employment status of people who required UPC. Regarding the psychiatric disorder diagnoses (according to the ICD- 9-CM) suffered by the subjects who required UPCs, we highlighted statistically significant differences between the two observation periods only in the ER subject sample (χ^2^ = 32.29; *p* = 0.001), in which we reported a significant reduction of subjects diagnosed with depressive disorder (SR = −2.09) during the COVID-19 period, as shown in Table 4. In the ER subject sample we reported another statistically significant difference between the two observation periods: patients with one or more medical comorbidities who requested UPCs were 737 (82.3%) in the pre-COVID-19 period and 579 (82.5%) in the COVID-19 period (Fisher’s exact = 0.000) (Table 4). We highlighted a statistically significant difference between the two observation periods regarding the previous treatments and care of the subjects who requested an UPCs both in ER (Fisher’s exact = 0.000) and CMHS (Fisher’s exact = 0.000), as reported in Table 4. In COVID-19 period, among the subjects who needed an UPC at ER we found a significant increase of people already treated at CMHS (SR = 6.32, *p* < 0.05) and a reduction of those being treated at Pathological Addiction Service (PAS) (SR = −2.42, *p* < 0.05), at private specialists (SR = −3.85, *p* < 0.05), at other or more services (SR = −2.97, *p* < 0.05) as well as of people not previously treated at any service or specialist (SR = −2.92, *p* < 0.05). Among subjects who needed an UPC at CMHS during COVID-19 period, we observed a significant increase of subjects in care at PAS (SR = 2.30, *p* < 0.05) and a reduction of people not previously treated at any service (SR = −2.32, *p* < 0.05) (Table 4). We did not highlight any statistically significant difference between the two observation periods regarding the use of alcohol and/or other substances in CMHS but only in ER, with a significant reduction of UPCs for people with alcohol and substance use (Table 4).

**TABLE 3:** Demographic characteristics of people who required UPC during the two observation periods.

Variables	People who required UPC in ER			People who required UPC in CMHS		
	Pre-COVID-19 period (*n* = 896)	COVID-19 period (*n* = 702)	Probability statistical test	Pre-COVID-19 period (*n* = 666)	COVID-19 period (*n* = 599)	Probability statistical test
**Gender, n (%)**						
Male	441 (49.2%)	357 (50.9%)	Not statistically significant	312 (46.7%)	272 (45.4%)	Not statistically significant
Female	455 (50.8%)	345 (49.1%)		355 (53.3%)	328 (54.6%)	
**Age, m ± DS**						
Years	43.87 ± 17.28	45.18 ± 18.0	Not statistically significant	45.98 ± 17.08	48.22 ± 15.68	*t* = −2.42 *p* = 0.015
**Nationality, n (%)**						
Italian	726 (81.0%)	549 (78.21%)	Not statistically significant	545 (81.8%)	503 (84.0%)	Not statistically significant
European (not Italian)	56 (6.3%)	53 (7.55%)		29 (4.4%)	27 (4.5%)	
Extra-European	114 (12.7%)	100 (14.2%)		92 (13.8%)	69 (11.5%)	
**Housing, n (%)**						
Parental family	215 (24%)	177 (25.2%)	χ^2^ = 15.35 *p* = 0.018 *SR = 2.41 **SR = −2.41	193 (28.9%)	156 (26.04%)	Not statistically significant
Marital family	242 (27%)	209 (29.8%)		213 (31.9%)	212 (35.4%)	
Alone	83 (9.2%)	73 (10.4%)		130 (19.5%)	133 (22.2%)	
Psychiatric facilities/communities	14 (1.6%)	24 (3.4%)*		41 (6.1%)	25 (4.1%)	
Other structures	75 (8.4%)	37 (5.3%)		46 (6.9%)	35 (5.8%)	
Homeless	18 (2%)	10 (1.4%)**		10 (1.5%)	4 (0.6%)	
Unknown	249 (27.8%)	172 (24.5%)		31 (4.6%)	32 (5.3%)	
**Employment status, n (%)**						
Unemployed	209 (23.3%)	172 (24.5%)	χ^2^ = 29.27 *p* = 0.000 ^§^ SR = 4.24 ^§§^ SR = −3.11	217 (32.6%)	189 (31.5%)	Not statistically significant
Employed	208 (23.2%)	182 (25.9%)		230 (34.5%)	227 (37.9%)	
Student	59 (6.6%)	31 (4.4%)		62 (9.3%)	37 (6.2%)	
Retired	62 (6.9%)	58 (8.3%)		71 (10.6%)	70 (11.7%)	
Disability pension	22 (2.5%)	48 (6.8%)^§^		28 (4.2%)	24 (4%)	
Unknown	336 (37.5%)	211 (30.1%)^§§^		21 (3.1%)	11 (1,8%)	

^§^, ^§§^, *, **, *** Standardized Residues (SR) with > or < 2 values and p < 0.05.

**TABLE 4:** Clinical characteristics of people who required UPC during the two observation periods.

Variables	People who required UPC in ER			People who required UPC in CMHS		
	Pre-COVID-19 period (*n* = 896)	COVID-19 period (*n* = 702)	Probability Statistical test	Pre-COVID-19 period (*n* = 666)	COVID-19 Period (*n* = 599)	Probability statistical test
**Psychiatric diagnosis (ICD-9-CM), n (%)**						
No previous psychiatric diagnosis	295 (32.9%)	259 (36.9%)	χ^2^ = 32.29; *p* = 0.001 §SR = −2.09	41 (6.2%)	39 (6.5%)	χ^2^ = 9.5744 *p* = 0.728 Not statistically significant
Schizophrenic spectrum disorders	99 (11%)	81 (11.5%)		162 (24.3%)	159 (26.5%)	
Bipolar disorders	55 (6.1%)	41 (5.8%)		45 (6.8%)	49 (8.2%)	
Depressive disorders	95 (10.6%)	53 (7.5%)§		103 (15.5%)	109 (18.2%)	
Anxiety disorders	50 (5.6%)	32 (4.6%)		58 (8.7%)	43 (8.2%)	
Personality disorders	118 (13.2%)	94 (13.4%)		90 (13.5%)	73 (12.2%)	
Alcohol/substance abuse and dependence	19 (2.1%)	22 (3.1%)		23 (3.5%)	15 (2.5%)	
Adjustment disorders	71 (7.9%)	69 (9.8%)		101 (15.2%)	75 (12.5%)	
Intellectual Disability	11 (1.2%)	14 (2.0%)		23 (3.2%)	15 (2.5%)	
Organic psychotic diagnosis	39 (4.4%)	29 (4.1%)		7 (1.1%)	9 (1.5%)	
Other	44 (4.9%)	8 (1.1%)		13 (2%)	13 (2.2%)	
**Alcohol/substance use**						
Absent	641 (71.5%)	546 (77.8%)*	Fisher’s exact = 0.000 *SR = 2.56 **SR = −2.83 ***SR = −2.28	575 (86.3%)	518 (86.5%)	Not statistically significant
Alcohol/Alcohol + substances	120 (13.4%)	83 (11.8%)**		12 (1.8%)	3 (0.5%)	
One or more substances	28 (3.1%)	38 (5.4%)		15 (2.3%)	17 (2.8%)	
Unspecified substance	69 (7.7%)	32 (4.6%)***		53 (8.0%)	57 (9.5%)	
Unknown	38 (4.2%)	3 (0.4%)		11 (1.7%)	4 (0.7%)	
**Medical comorbidity, n (%)**						
Present	737 (82.3%)	579 (82.5%)	Fisher’s exact = 0.000	205 (30.8%)	191 (31.9%)	Not statistically significant
Not present	119 (13.3%)	120 (17.1%)		443 (66.5%)	401 (66.9%)	
Unknown	40 (4.4%)	3 (0.4%)		18 (2.7%)	7 (1.2%)	
**Previous treatments and care, n (%)**						
CMHS	373 (41.6%)	404 (57.5%)^§^	Fisher’s exact = 0.000 ^§^ SR = 6.32	429 (64.1%)	426 (71.1%)	Fisher’s exact = 0.000 ^§§^ SR = 2.30
PAS	24 (2.7%)	7 (1.0%)		5 (0.8%)	14 (2.3%)^§§^	
Other private specialists	42 (4.7%)	9 (1.3%)		9 (1.4%)	11 (1.8%)	
Other or more than one outpatient service	50 (5.5%)	18 (2.6%)		18 (2.7%)	9 (1.5%)	
No previous treatment and care	402 (44.9%)	264 (37.6%)		188 (28.2%)	136 (22.7%)	
Unknown	5 (0.6%)	0 (0.0%)		17 (2.6%)	3 (0.5%)	

^§^, ^§§^, *, **, *** Standardized Residues (SR) with > or < 2 values and p < 0.05.

### Multiple Logistic Regression Between Urgent Psychiatric Consultations and Selected Variables

We analyzed in multiple logistic regression (backward and forward stepwise model), the dependent variable (“UPCs in the pre-COVID-19 period = 0,” “UPCs in the COVID-19 = 1”) and all other selected variables (independent variables) both in the ER and CMHS UPC sample. Regarding the UPC carried out in ER (Table 5), we reported the following variables with a statistically significant association: the month of March, previous treatments and care (in PAS, other specialists and other or more than one outpatient service as well as no previous treatment and care) and many psychiatric diagnoses with a < 1 Odds Ratio, whereas referral to UPCs from psychiatric facilities/communities and UPC outcome (referral to CMHS, to more than one outpatient service, other) with > 1 Odds Ratio. Regarding the UPC at CMHS, we highlighted the following statistically significant associations (Table 6): Extra-European nationality, previous treatments and care at more than one outpatient service and the psychiatric diagnosis of Intellectual Disability with < 1 Odds Ratio, whereas UPC setting in Video and Telephone call, an absent medical comorbidity and the UPC outcome of home visit with a > 1 Odds Ratio.

**TABLE 5:** Significant variables at backward and forward stepwise multiple logistic regression (dependent variable: UPCs in ER).

Independent variables	Odds ratio	95% confidence interval	Probability
**Month**			
March	0.49	0.30; 0.81	*p* = 0.006
**Referral to UPCs**			
Psychiatric facilities/communities	2.71	1.69; 4.36	*p* = 0.000
**Previous treatments and care**			
PAS	0.15	0.07; 0.33	*p* = 0.000
Other specialists	0.16	0.08; 0.32	*p* = 0.000
Other or more than one outpatient service	0.20	0.12; 0.33	*p* = 0.000
No previous treatment and care	0.33	0.23; 0.46	*p* = 0.000
**Psychiatric diagnosis**			
Schizophrenic spectrum disorders	0.43	0.25; 0.75	*p* = 0.003
Bipolar disorders	0.34	0.22; 0.52	*p* = 0.000
Depressive disorders	0.33	0.20; 0.53	*p* = 0.000
Anxiety disorders	0.26	0.17; 0.41	*p* = 0.000
Personality disorders	0.30	0.18; 0.50	*p* = 0.000
Alcohol/substance abuse and dependence	0.36	0.24; 0.54	*p* = 0.000
Intellectual disability	0.49	0.32; 0.75	*p* = 0.001
Organic psychotic diagnosis	0.09	0.03; 0.27	*p* = 0.000
Other	0.42	0.20; 0.89	*p* = 0.023
**UPC outcome**			
Referral to CMHS	1.33	1.05; 1.68	*p* = 0.017
Referral to more than one outpatient service	3.11	1.92; 5.05	*p* = 0.000
Other	2.26	0.13; 0.54	*p* = 0.000

**TABLE 6:** Significant variables at backward and forward stepwise multiple logistic regression (dependent variable: UPCs in CMHS).

Independent variables	Odds ratio	95% confidence interval	Probability
**Nationality**			
Extra-European	0.72	0.53; 0.97	*p* = 0.030
**UPC setting**			
Video call	2.21	1.55; 3.16	*p* = 0.000
Telephone call	5.48	3.49; 8.60	*p* = 0.000
**Previous treatments and care**			
More than one outpatient service	0.31	0.17; 0.55	*p* = 0.000
**Psychiatric diagnosis**			
Intellectual disability	0.57	0.33; 0.99	*p* = 0.046
**Medical comorbidity**			
Absent	1.21	1.00; 1.46	*p* = 0.046
**UPC outcome**			
Home visit	5.88	1.62; 21.28	*p* = 0.007

## Discussion

This observational retrospective study focused on the evaluation of COVID-19 pandemic impact on psychiatric emergencies in order to highlight the effects of such an upheaval on the mental health of individuals and on the organization of mental health services. Moreover, this study investigated two different settings where urgent psychiatric consultations are normally carried out in order to illustrate which mental health organization presented the most impressive change during the pandemic period. We observed that in both settings of UPCs the number of consultations per month differed from that of the previous year, showing a sort of relationship with the pandemic progress and its consequences on society. During the month of March 2020, in ER, we reported a drastic reduction of UPCs, which then increased in the following months, in line with most studies in different countries (30–36, 48). The small number of consultations could be explained by the reduction of other ER activities to focus on pandemic needs and by decreased demand for UPC by the population due to the fear of contagion in hospital. On the contrary, in CMHS, more UPCs were carried out during the month of March 2020 and the number of UPCs per patient was significantly higher during the pandemic period in comparison to the previous period, probably due to the reduction of hospitalizations, as a recent study has pointed out (35). CMHS represented a safe place for emergencies during the environmental crisis, offering treatments and care even during the outbreak and related restrictive measures, despite profound changes to its work organization: most programmed activities were performed remotely through telephone or video calls; group activities and Day-Hospital were suspended; admissions to psychiatric facilities were extremely reduced (38, 46, 49). In any case, the general population not being able to receive adequate psychological and relational support may have caused the increase in UPC request in CMHS. In this regard, our study confirms the statistically significant difference in settings of UPCs in CMHS in comparison with the previous year due to the more frequent use of telephone and video calls during the pandemic. In fact, the psychiatrists and the nurses of CMHS had to change their modality of work, assessing the psychological state of patients and the need for urgent treatments through telephone and/or video calls, in order to guarantee them appropriate treatments and care. Around the world, the pandemic has disrupted the traditional “face to face” evaluation and, thanks to the COVID-19 pandemic, telemedicine has become a tool to ensure safe care also in psychiatry (28). Some studies showed the utility of tele-psychiatry in contexts of humanitarian emergencies, emphasizing both its effectiveness and good applicability (29). A recent review has pointed out the consistent diagnostic reliability, satisfactory clinical outcomes and patient satisfaction linked to tele-psychiatry (50). Nevertheless, the effectiveness of telemedicine is conflicting, because it can be an obstacle to the therapeutic alliance (28) and can be difficult to use by people with visual or hearing impairments or by those suffering from severe problems related to psychotic disorders (51).

Both in ER and CMHS, most subjects who required UPCs came from home during the two observation periods, but, during the pandemic, more patients who lived in psychiatric facilities required UPCs in ER. Similarly, more individuals from other institutions (like jail, other communities, etc.) were referred to CMHS for UPCs. These observations put in evidence that people previously suffering from severe psychiatric disorders, who had required residential care, were more vulnerable to the social upheaval caused by the COVID-19 outbreak. According to many authors, patients in psychiatric facilities were more affected than others by restrictive measures and social distancing during the pandemic, being confined without any possibility of movement and social interactions and with the fear of being abandoned (8, 24, 37).

During the pandemic, we observed a significant increase of two clinical reasons for UPC in ER: aggressiveness and socio-environmental maladjustment, which could represent a reaction to the restrictive measures imposed by government authorities, measures often poorly tolerated (16). In some cases, the measures of home confinement caused an increase in family tensions and domestic violence, especially in contexts where aggressive behavior was already present (18). Another effect of the restrictive measures was the reduction of employment and, above all, the bankruptcy of companies and industries which led to the dismissal of many people and the consequent financial crises in many families. In ER, the more representative clinical reasons for UPCs, aggressiveness, socio-environmental maladjustment and psychiatric symptoms in organic disorder, were more frequent in some months: April, May, July, November and December. This trend seems to follow the phases of COVID-19 pandemic in Italy, where the first epidemic peak ended in May 2020 and the second one started in October 2020 (52), suggesting a direct correlation between pandemics and psychic reactions. Regarding the UPCs in CMHS during the pandemic, we reported a significant increase of two clinical reasons: psychopharmacological therapy control, which could be a consequence of reduced routine outpatient activity, and state of mania, which could represent one of the most frequent psychiatric emergencies, sometimes related to organic conditions or substance abuse (53) or to COVID-19 infection (54). Other authors reported an increase of substance use during the COVID-19 period, especially in relation to isolation measures, which we did not find (17, 36). Similarly to our observations in ER, the progression of clinical reasons for UPCs in CMHS reflects the evolution of the pandemic: in March, in concomitance with the first lockdown, the most frequent request was represented by the control of psychopharmacological therapy and, in November 2020, at the beginning of the second lockdown, we reported an increase of UPCs for socio-environmental maladjustment.

Our study highlighted a significant reduction of UPCs for anxious-depressive states in CMHS during the pandemic period, in particular during the month of November, confirming our previous research (38, 46). The reduced UPCs for depressive disorders, in line with some studies (35, 40) but in contrast with others (8, 19, 21, 22), could apparently be countertrend and partially explainable. It could be conditioned by the relatively short time from the outbreak. In fact, as predicted by some authors in analogy with other socio-health emergencies (55, 56), we could report over time an increase of anxiety and depressive disorders as well as of post-traumatic stress disorder and anticonservative behavior only at the end of the health and social emergency caused by COVID-19 outbreak. Consistent with this data, during the pandemic period the individuals with depressive disorders required less frequent UPCs in ER in comparison with the previous period. This result is confirmed by several studies carried out in ER (27, 32, 35) and probably outlines people who, already suffering from a depressive disorder before the pandemic, may have experienced a paradoxical sense of stability and balance from the application of restrictive measures due to the reduction of environmental stimuli. Alternately, they could have benefited from the help offered by telemedicine (35, 37). Nevertheless, this data is in contrast with other studies which reported an increase in emergency access for depressive and anxiety disorders during the pandemic period (19–22, 41) and suicide rate (57, 58). Furthermore, our research did not report any increase of self-harm behavior or suicide attempts as reasons for UPCs during the pandemic as highlighted by some studies conducted after a short period from the start of the pandemic (59–61). Older studies show, however, that in the early stages of emergencies, such as wars or profound social crises, there may be a reduction in suicidal behavior, probably in relation to the fact that the survival instinct prevails over the collective danger of infection (62, 63).

Regarding the outcomes of UPCs in ER, we observed a reduction of referral to domicile and to General Practitioner and an increase of referrals to CMHS and other services during the pandemic period compared to the previous one. In particular, sending to the CMSH and other services was prevalent in September and December of the pandemic period, suggesting that community psychiatric services had been able to reorganize, ensuring an adequate level of care after a few months from the outbreak of the pandemic (49). Among the outcomes of UPCs in CMHS, the referral to home care was significantly prevalent in the pandemic period in concomitance with the reduction of outpatient activities due to restrictive measures, in particular during the month of June, a few months after the beginning of the Italian lockdown.

Regarding the clinical activities during UPCs, we observed no differences with the previous period in UPC activities carried out in ER, whereas we reported a statistically significant reduction in prescription and administration of psychopharmacological therapy during the pandemic period in UPCs carried out in CMHS, probably related both to the reduced clinical activities concomitant to the restrictive measures and the increase in UPCs carried out remotely, as noted by other authors (64). In ER during the pandemic period, we appreciated the more frequent requests for UPCs from people who lived in psychiatric facilities and/or communities and a reduction from homeless people, in a statistically significant way compared to the previous period, suggesting the different but equally negative impact of social isolation due to the outbreak pandemic on the most vulnerable people, as highlighted by other authors (24, 37). The effects of restrictive measures in the pandemic period could also justify the significant increase of UPC request in ER from people with disability pensions, confirming the fragility of these individuals. The demographic characteristics of individuals who required UPC in CMHS were similar between the two periods with only one statistically significant difference: people visited in UPC during pandemic period were older than those in the previous period. Some recent studies have pointed out that older population encountered greater difficulties in adapting to new modalities of assistance provided by community outpatient psychiatric services (25). The forced isolation during the first lockdown, induced stress, anxiety and phobic behavior especially in older people (45). Furthermore, vulnerability to the biological, psychological and environmental consequences of the COVID-19 pandemic and, at the same time, the fear and stress related to a possible contagion would increase with increasing age (65).

In the pandemic period, we appreciated an increased request for UPCs in ER from subjects already treated at CMHS, suggesting the negative psychological consequences of the COVID-19 pandemic in people already suffering from a psychiatric disorder who manifested great clinical and social vulnerability (8, 66). The increase in requests from these subjects could also be justified by the reduction or reorganization of clinical activities in CMSH. Similarly, in CMHS we observed an increase of UPC requests from subjects who were already in care of PAS, probably due to the reduction in activity in drug addiction services. The presence of medical comorbidity appeared to be significantly less frequent only among the UPCs in ER during the pandemic period. This data contrasts with other observations in the literature which described an increase in UPC for subjects with medical pathologies (65). In addition, we observed a reduction of UPCs in ER for alcohol and substance use during pandemic, in contrast with some studies which show an increase in consultations carried out for subjects with substance abuse in the pandemic (31, 37, 67).

Our model of multiple logistic regression is consistent with almost all of our other results. Regarding UPCs in ER, we highlighted that living in a psychiatric facility or community is a factor associated with an increase of UPC during pandemic but not suffering from most psychiatric disorders or being treated in outpatient service, which, on the contrary, represent detrimental factors for UPC in outbreak. These results further identify in people already affected by a severe mental disorder the population most psychologically vulnerable to socio-environmental distress induced by COVID-19, as highlighted by other authors (68). We confirmed that the month of March 2020 was a detrimental factor for the number of UPCs in ER. In fact, in this month the first peak of widespread epidemic occurred in Italy and generated social alarm for the risk of infection, requiring dramatic changes in health organizations, especially in Emergency Departments, and social isolation with the beginning of Italian lockdown. This result highlights the reduced request for psychiatric intervention in a period of socio-environmental urgency, in which the priority was relative to the survival of the individual. Regarding UPCs in CMHS, our regression model confirmed that the most representative settings were telephone and video calls and patients who did not present a medical comorbidity were associated with an increased number of UPC in CMHS during the pandemic period. On the contrary, patients affected by Intellectual Disability or treated by more than one outpatient service were detrimental factors for UPC in CMHS during COVID-19 as well as non-European nationality. This last data could indicate a potential marginalization of immigrant groups who have less access to care, as reported by some authors (69). Our regression model highlighted the outcomes more frequently associated with UPCs during COVID-19 period: referral to outpatient service from UPC in ER and home care from UPC in CMHS, neither indicating hospitalization, which was drastically reduced during the first months after pandemic outbreak (70). In any case, as observed by an author (71), we will appreciate the real impact and consequences of the COVID-19 pandemic on mental health of the general population only in future.

### Limitations and Advantages

The limitations of this study include the observational-retrospective design, which does not allow us causal inferences. First, we put in evidence that for various variables, especially demographic and clinical, it was not always possible to find complete data for “Employment status” of people included in our ER sample and “previous treatment and care” in Pathological Addiction Service, for example. Regarding the consultations carried out in CMHS, we could not collect the data relating to February 2021 due to the recent change of the informatics application, which did not allow us to collect complete data, therefore we restricted our analysis to 11 months. Furthermore, the monocentric design of this study limits the comparison with other services and therefore the generalizability of our results. The advantages of this study include the period of collection and analysis which is parallel to the emergency period of the pandemic. In fact, our study has permitted us to promptly obtain early data on the evolution of the COVID-19 pandemic, providing a detailed picture of real settings. Among the advantages of the study, we also report the size of the sample, the length of the observation period, the comparison with both the corresponding pre-COVID-19 period and two emergency settings, which gave us an exhaustive picture of the activities in ER and CMHS during pandemic.

## Conclusion

This study highlights an initial and significant reduction in urgent psychiatric consultations in ER during the beginning of lockdown for the containment of COVID-19 infection and a concomitant increase in urgent consultations carried out in CMHS, testifying that community outpatient service continued to guarantee treatments, care and support despite the reorganization and reduction of activities due to the pandemic. Specifically, all the consultations in CMHS were carried out through the modality of tele-psychiatry (telephone and video calls), which in fact inaugurated a new way of contacting patients in a situation of environmental crisis during COVID-19 pandemic. The increase of UPCs in CMHS identify in the outpatient service a safe and accessible place where support is available, especially for the most fragile and vulnerable individuals. Compared to the pre-COVID-19 period, the relevant clinical reasons for psychiatric emergencies in ER were represented by aggressiveness and socio-environmental maladjustment, both expressions of difficulties in adapting to the dramatic pandemic which imposes drastic changes in lifestyle. The need for a modification of psychopharmacological therapy and the states of mania prevailed among the UPC clinical reasons at CMHS during the pandemic, further indicating the different populations addressed to ER and CMHS and the activities performed in the two settings. During the pandemic period, the subjects suffering from a severe psychiatric disorder, who had required residential treatment in a psychiatric facility or community or people with disability pension represented the most vulnerable population for clinical and social needs worsened by pandemic crisis. In light of our findings, we conclude by emphasizing that the most vulnerable people, who need constant support from service, or people affected by disability, more frequently required attention and care both in ER and CMHS, whereas other marginalized people, such as the homeless or immigrants, remained isolated without asking for support from health service. In order to reduce the impact of the COVID-19 pandemic on mental health, psychological support interventions for the general population should be implemented, having particular regard for more vulnerable and psychologically fragile people.

## Data Availability Statement

The raw data supporting the conclusions of this article will be made available by the authors, without undue reservation.

## Ethics Statement

The studies involving human participants were reviewed and approved by the Ethics Committee of the Area Vasta Emilia Nord (protocol AOU 0016106/20; protocol no. 0035176/20 and protocol no. 0035169/20 of 03/12/2020) and authorization of the Modena AUSL, Decree no. 1381 of 22-6-20. The patients/participants provided their written informed consent to participate in this study.

## Author Contributions

RDL and MP designed the research study and analyzed the data. DB, AC, DD, CE, RF, GF, FL, SP, and LV performed the research. SR and PF provided help and advice on the research. RDL and PF wrote the manuscript. All authors contributed to editorial changes in the manuscript, read, and approved the final manuscript.

## Conflict of Interest

The authors declare that the research was conducted in the absence of any commercial or financial relationships that could be construed as a potential conflict of interest.

## Publisher’s Note

All claims expressed in this article are solely those of the authors and do not necessarily represent those of their affiliated organizations, or those of the publisher, the editors and the reviewers. Any product that may be evaluated in this article, or claim that may be made by its manufacturer, is not guaranteed or endorsed by the publisher.

## Abbreviations

ER: Emergency Rooms
CMHS: Community Mental Health Services
UPCs: Urgent Psychiatric Consultations
PAS: Service of Substance Use.
